# Assessing differential attrition in clinical trials: self-monitoring of oral anticoagulation and type II diabetes

**DOI:** 10.1186/1471-2288-7-18

**Published:** 2007-05-02

**Authors:** Carl Heneghan, Rafael Perera, Alison Ward A, David Fitzmaurice, Emma Meats, Paul Glasziou

**Affiliations:** 1 Department of Primary Health Care, University of Oxford, Oxford, UK; 2 Department of Primary Health Care, University of Birmingham, Birmingham, UK

## Abstract

**Background:**

Analyzing drop out rates and when they occur may give important information about the patient characteristics and trial characteristics that affect the overall uptake of an intervention.

**Methods:**

We searched Medline and the Cochrane library from the beginning of the databases to May 2006 for published systematic reviews that compared the effects of self-monitoring (self-testing) or self-management (self-testing and self-dosage) of oral anticoagulation or self-monitored blood glucose in type 2 diabetics who were not using insulin. We assessed all study withdrawals pre-randomization and post randomization and sought information on the reasons for discontinuation of all participants.

To measure the differential between groups in attrition we used the relative attrition (RA), which is equivalent to relative risk but uses attrition as the outcome (i.e. attrition in intervention group/attrition in control group). We determined the percentage drop outs for control and intervention groups and used DerSimonian and Laird random effects models to estimate a pooled relative attrition. L'abbe type plots created in R (version 2.0.2) were used to represent the difference in the relative attrition among the trials with 95% confidence areas and weights derived from the random effects model.

**Results:**

With self-monitoring of blood glucose in type 2 diabetes, attrition ranged from 2.3% to 50.0% in the intervention groups and 0% to 40.4% in the control groups. There was no significant difference between the intervention and control, with an overall RA of 1.18 [95% CI, 0.70–2.01]. With self-monitoring of oral anticoagulation attrition ranged from 0% to 43.2% in the intervention groups and 0% to 21.4% in the control group. The RA was significantly greater in the intervention group, combined RA, 6.05 [95% CI, 2.53–14.49].

**Conclusion:**

This paper demonstrates the use of relative attrition as a new tool in systematic review methodology which has the potential to identify patient, intervention and trial characteristics which influences attrition in trials.

## Background

Loss to follow up after recruitment and attrition in randomized controlled trials affects the generalisability of the conclusions. [1,2] Loss to follow up occurs when participants' information cannot be obtained for one reason or another, whereas attrition is the exclusion or drop-out of individuals for a particular reason after randomization to the intervention or control group. [2] Attrition forms one of the four predominant biases in clinical trials: selection, performance, attrition and detection bias. Investigators frequently exclude patients from trial analyses, most commonly because of ineligibility or protocol violations. Trials that exclude more patients tend to be larger and published earlier than those that do not. [2]

Analyzing drop out rates and when they occur may give important information about the patient characteristics and trial characteristics that affect the overall uptake of an intervention. It is equally important to assess the inclusion and exclusion criteria's impact on subsequent drop out rates and perhaps the most significant effect is observed by the assessment of the comparative losses between the control and intervention groups. [1]

To analyse the impact of relative attrition on results of systematic reviews we considered self-monitoring of oral anticoagulation and oral diabetes as the rates of attrition in these published systematic reviews vary considerably. On average 22% of patients assigned to self-monitoring of oral anticoagulation were unable to complete the intervention, range of 9 – 43%. [3] For type 2 diabetics receiving oral therapy, self-monitoring, withdrawals have been reported as high as 50%. [4,5] We aimed to analyse the drop out rates from randomized controlled trials of self monitoring of oral anticoagulation and oral diabetes, to examine the patient and trial characteristics that effect attrition. In addition we aimed to analyse the relative difference between the intervention and control groups using a measure of relative attrition.

## Methods

We searched Medline and the Cochrane library from the beginning of the databases to May 2006 for published systematic reviews that compared the effects of self-monitoring (self-testing) or self-management (self-testing and self-dosage) of oral anticoagulation or self-monitored blood glucose in type 2 diabetics who were not using insulin. MeSH terms used were "anticoagulants", "vitamin-K" OR "coumarins" AND "consumer-participation" OR "self-care" OR "self-administration". For diabetes terms used were "diabetes mellitus adult-onset" OR "diabetes mellitus" OR "non insulin dependent" OR "diabetes mellitus type II" OR "NIDDM" AND "self-care" OR "self-administration" OR "blood glucose self monitoring". In addition we used the systematic review filter "systematic". From these reviews we obtained the full-text papers of the included randomized controlled trials. We also repeated the search strategies of the systematic reviews to search for recently published randomized controlled trials in both areas.

### Data abstraction

We assessed all studies for inclusion and exclusion criteria and for study withdrawals before randomization and post randomization. We extracted information on disease characteristics and the training undertaken in the intervention and control groups. We extracted descriptors on the population studied, including the number of participants who refused or were excluded from entering the trial. We sought information on the reasons for discontinuation of all participants allocated to the intervention and the control. Where data was insufficient we wrote to authors for clarification.

### Data analysis

To measure the differential between groups in attrition we used the relative attrition (RA), which is equivalent to relative risk but uses attrition as the outcome (i.e. attrition in intervention group/attrition in control group). A relative attrition of one means the attrition in both the intervention and control were equivalent; less than one, attrition is less in the intervention than the control arm, and greater than one, attrition in the intervention group was higher. With this measure we can estimate an average RA and detect trials with lower or higher than average RA, and look for trial characteristics that account for this effect.

We determined the percentage drop outs for control and intervention groups. Due to the heterogeneity in drop out rates between trials we used the DerSimonian and Laird random effects models to estimate a pooled relative attrition for all trials using STATA (version 8.2). Data was entered by two reviewers independently and checked. L'abbe type plots and Forest plots created in R (version 2.0.2) were used to represent the difference in the relative attrition among the trials with 95% confidence areas and weights derived from the random effects model. Heterogeneity was examined using chi-squared and I-squared statistics and where possible we used meta-regression to test for the effect of trial characteristics on attrition.

## Results

For self-monitoring of blood glucose in patients with type two diabetes, we identified five [6-10] reviews, and included eleven randomized trials (table 1), [4,5,11-19] comprising 1,689 participants. For oral anticoagulation we identified four reviews [3,20-22] and included 16 randomized trials (table 2), [23-38] comprising 3,788 participants.

**Table 1:** Diabetes study characteristics

Source	Inclusion Criteria	Duration of study mths.	Nos. assigned to Intervention & (%) drop out	Nos. assigned to Control & (%) drop out	Intervention for self-monitoring group	Control group intervention
^19 ^Wing 1986, US	NIDDM, ≥ 20% above ideal weight. Use oral hypoglycaemic medication or insulin.	14	25 (8.0)	25 (12.0)	Taught to make changes in diet and exercise if SMBG elevated.	Standard behavioural weight control treatment
^14 ^Fontbonne 1989, France	NIDDM, no rapidly progressing diabetic complications and no severe illness.	6	68 (17.6)	68 (20.6)	All patients had pre entry visit, and training in SMBG.	Usual diabetic clinic care
^11 ^Allen 1990, US	NIDDM, No prior experience of monitoring	6	31 (12.9)	30 (10.0)	Diet and exercise counselling. Individual instruction for SMBG	Diet and exercise counselling. Individual instruction for SMUG
^13 ^Estey 1990, Canada	NIDDM On diet or oral hypoglycaemic drugs.	3	30(6.7)	30 (16.7)	3 day education program (test and control). Inc. nurse, dietician, social worker and pharmacist.	Same 3-day education programme
^5 ^Rutten 1990, Holland	Treated for NIDDM for least 6 months. Not taking insulin. Not under treatment for other conditions.	12	66 (50)	83 (12.0)	Given instruction on SMBG on 2–5 occasions. Given advice and therapeutic goals.	Usual General Practice care
^15 ^Gallichan 1994, UK	NIDDM, on oral hypoglycaemic agents.	6	15 (33.3)	12 (16.7)	Instruction on SMBG.	Instruction on SMUG
^19 ^Muchmore 1994, US	NIDDM treated with diet alone/diet + oral sulfonylurea hypoglycemic agents. No use of self monitoring for 3 months.	7	15 (20.0)	14 (21.4)	Training in SMBG from nurse educator individually and in groups	General strategies of diabetes control, exercise, recommended by ADA.
^17 ^Miles 1997, UK	Newly diagnosed NIDDM	6	68 (18)	23 (28.0)	Group education, within a week of diagnosis. 4 education sessions supervised by nurse. Individual SMBG techniques checked at 1 month.	Group education, within a week of diagnosis. 4 education sessions supervised by nurse. Individual SMUG technique checked at 1 month.
^18 ^Schwedes 2002, Germany	NIDDM, BMI > 25 kg/m2. Treated with diet or diet in combination with sulfonylureas or metformin.	6	125 (9.6)	125 (12.0)	Standardized counselling and instruction on use of SMBG	None standardised counselling on diet and lifestyle.
^4 ^Guerci 2003, France	NIDDM, no prior experience of monitoring and able to carry out self monitoring.	6	345 (47.5)	344 (40.4)	Training in SMBG by GP	Usual General Practice care
^12 ^Davidson 2005, US	NIDDM, on entering Diabetes Managed Care Program.	6	43 (2.3)	45 (0)	Training in SMBG by specialist nurse	Diabetes Managed Care Program.

SMBG self-monitoring of blood glucose

SMUG self-monitoring of urine glucose

**Table 2:** Oral anticoagulation study characteristics

Source	Inclusion Criteria	Duration of study mths.	Nos. assigned to Intervention & (%) drop out	Nos assigned to Control & (%) drop out	Intervention for self-monitoring group	Control group intervention
^38 ^White 1989, US	Inpatients with a duration of OAT of at least 8 weeks §	2	26 (11.5)	24 (4.2)	Management by general internists	ACC
^29 ^Horstkotte 1998, Germany	Outpatients with the St Jude Medical prosthesis ‡	N/A	75 (1.3)	75 (0)	INR twice a week and contact clinic by phone	PCP
^34 ^Sawicki 1999, Germany	Any indication for anticoagulation and life long treatment ‡	6	90 (14.4)	90 (7.8)	3 educational sessions. SM	PCP
^23 ^Beyth 2000, US	Inpatients §	6	163 (22.1)	162 (0)	1-hour education session and contact clinic by phone	PCP
^24 ^Cromheecke 2000, Holland	Long term OAT at least 6 months treatment ‡	3	101 (0)	100 (0)	2-educational sessions, SM	ACC
^32 ^Kortke 2001, Germany	Patients after mechanical heart valve surgery ‡	24	305 (8.9)	295 (21.4)	Trained in self-monitoring 6–11 days after operation	PCP
^35 ^Sidhu 2001, UK	Patients after mechanical heart valve surgery ‡	24	51 (33.3)	49 (2.0)	2-educational sessions, SM	PCP
^25 ^Fitzmaurice 2000, UK	Long term OAT at least 6 months treatment ‡	6	30 (23.3)	26 (0)	2-educational workshops, SM	PCP
^27 ^Gadisseur 2003, Holland	Long term OAT at least 3 months treatment ‡	6	99 (19.2)	161 (0)	3 educational sessions. SM by telephone	ACC
^28 ^Gardiner 2004, UK	Long term OAT At least 8 months ‡	6	44 (43.2)	40 (2.5)	2-educational sessions 1 week apart	ACC
^31 ^Khan 2004, UK	At least 12 months OAT with AF. Age > 65 yrs ‡	6	44 (9.1)	41 (4.9)	2 hour education session, contact by phone	ACC
^36 ^Sunderji 2004, Canada	OAT for at least 1 month ¤	8	70 (28.6)	70 (0)	2-educational sessions, SM	PCP
^33 ^Menendez-Jandula 2005, Spain	OAT for at least 3 months therapy ‡	11.8	368 (21.5)	369 (2.4)	2-educational sessions, taught by nurse. SM	ACC
^37 ^Voller 2005, Germany	long term OAT with non valvular AF ‡	5	101 (19.8)	101 (0)	Standard training course of 3 sessions	PCP
^26 ^Fitzmaurice 2005, UK	Unselected patients in a general practice population	12	337 (41.5)	280 (13.2)	2-educational sessions, taught by nurse. SM	PCP or ACC
^30 ^Katz Unpublished, US	Long term OAT attending anticoagulation clinic ‡	12	101(0)	100(0)	All patients were trained by nurse, video, and tested with a skills and knowledge checklist prior to randomization	ACC

§ Coumatrack Monitor ‡ Coagucheck System ¤ Pro time Microcoagulation system ACC: Anticoagulation Clinic Care PCP: Primary Care physician managed SM: self adjusted treatment

**Figure 1:**
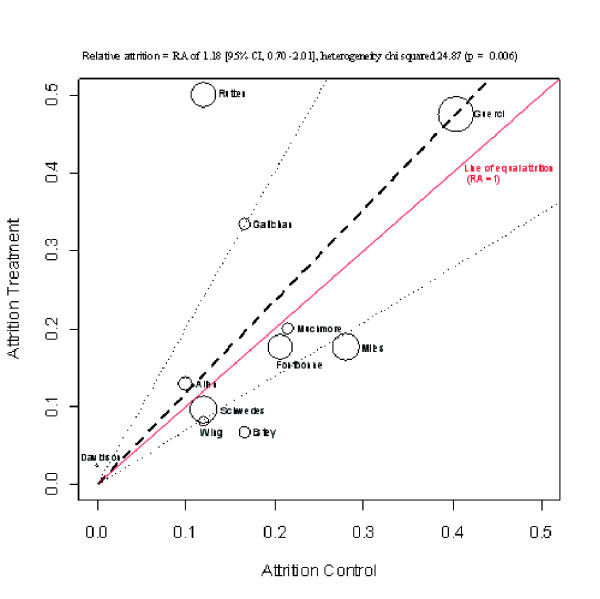
Diabetes attrition L'abbe plot.

With self-monitoring of blood glucose in type 2 diabetes (table 1), attrition ranged from 2.3% [12] to 50.0% [5] in the intervention groups and 0% [12] to 40.4% [4] in the control groups. There was no significant difference between the intervention and control, with an overall RA of 1.18 (95% CI, 0.70 -2.01), heterogeoneity chi squared 24.87 (p value = 0.006), I-squared 59.8%. (figure 1)

Two of the included studies were considered to be of high quality [11,12] in the Welschen systematic review and two studies [4,18] had a significant effect of SMBG on HbA1c results. Attrition was not significantly related to study duration. Seven trials had less than 100 participants, [11-13,15-17,19] and one trial of 689 participants [4] accounted for 41% of the patients studied. Study size as well as the year of the study revealed no significant trends in terms of attrition. Table 3 gives the reasons for drop outs stated by the trial author.

**Table 3:** Drop out reasons in trials of Diabetes

Source	Reasons Given for drop out in the intervention and control group
^19 ^Wing 1986, US	Moved out of area, patient withdrawal and exclusion.
^14 ^Fontbonne 1989, France	Lost to follow up, no reasons given
^11 ^Allen 1990, US	Inappropriate randomization and drop outs for unknown reasons
^13 ^Estey 1990, Canada	Hospitalizations, initiation of insulin therapy, one death and failure to keep clinic appointments
^5 ^Rutten 1990, Holland	Unwilling or incapable of self-monitoring, death, referral to internist, failure to adhere to protocol, moved out of the area and admission to hospital
^15 ^Gallichan 1994, UK	Failure to present for re-testing
^19 ^Muchmore 1994, US	No reasons given
^17 ^Miles 1997, UK	Refusal to change over to alternative strategy, patients found the monitoring too stressful, conversion to insulin, moved out of area, found not to have diabetes post randomization and protocol violations
^18 ^Schwedes 2002, Germany	No reasons given
^4 ^Guerci 2003, France	Adverse events, patient non-compliance, consent withdrawal, loss to follow up, death, protocol violation, lack of information and other reasons
^12 ^Davidson 2005, US	Patient failed to return for follow up appointment.

**Figure 2:**
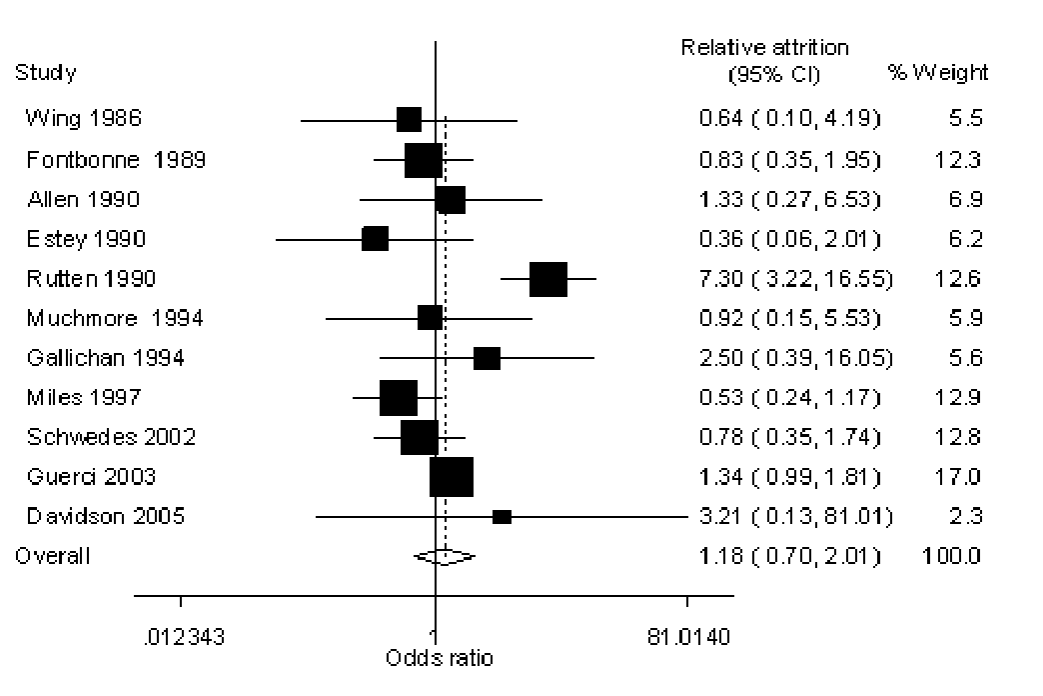
Diabetes attrition Forest plot.

Two studies report a RA smaller than the lower limit of the 95% CI, equivalent to a higher than expected attrition in the control group (figure 2). Of these two, the Estey trial [13] control group received a standard 3-day educational session. A comparison between those who dropped out and those who remained in the study did not indicate any significant differences with respect to baseline demographics. The second (Miles) study [16] invited all newly diagnosed diabetics attending a patient education programme, participants were randomly allocated to blood glucose or urinary glucose.

Three studies report a RA higher than the upper limit of the 95% CI, equivalent to higher than expected attrition in the intervention group. In the Gallichan study [15] most patients preferred urine testing (71%), and 33.3% dropped out of the intervention group. The Davidson trial [12] recruited patients in a community clinic on entering a diabetes managed care program. Only one patient did not return to see the nurse or dietician after randomization. In the Rutten trial [5] set in eight general practices, only 50% of the treatment group proved able to carry out accurate self-monitoring. Patients under 40 and older than 75 years of age were excluded, as were patients with co-morbid diseases under the care of a hospital.

In the largest trial of 689 participants, [4] the attrition was high at 47.5% relative to 40.4% in the control group RA, 1.18 (95% CI, 0.99–1.39). This is the only trial where participants received training in SMBG by the general practitioner. Of note 299 patients were not able to provide two HbA1c measurements in a two month run in period post randomization, and were removed from the study. The remaining 689 patients were asked to perform at least 6 capillary assays per week (3 different days per week including weekends). A further 303 patients dropped out of the study, of these 240 had a reason reported for discontinuation: adverse event (n = 6), patient non-compliance (n = 33), consent withdrawal (n = 15), patient lost to follow up (N = 19), death (n = 4), protocol violation (n = 21), lack of information on patients (n = 92), and other reasons as stated in the paper (n = 110).

### Self-monitoring of oral anticoagulation

**Figure 3:**
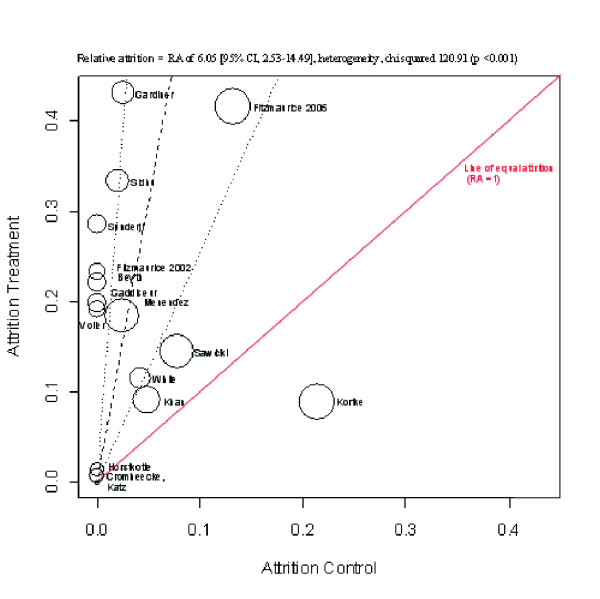
Oral anticoagulation attrition L'abbe plot.

With self-monitoring of oral anticoagulation (table 2) attrition ranged from 0% [24] to 43.2% [28] in the intervention groups and 0% [23-25,27,29,30,36,37] to 21.4% [32] in the control group. The RA was significantly greater in the intervention group, combined RA 6.05 (95% CI, 2.53–14.49), heterogeneity chi squared 120.91 (p value < 0.001), I-squared 88.4%.(figure 3)

Four of the included studies were judged to be of low quality [28,31,32,38] in the Heneghan systematic review. Study duration ranged from two months [38] to two years, [32,35] four trials had less than 100 participants [25,28,31,38] and three trials [26,32,33] accounted for 51% of the patients studied. Study size as well as the year of the study revealed no trend in terms of attrition. In addition attrition was not significantly related to study duration. Furthermore analysis of self-management versus self-testing only, showed no significant difference in relative attrition (meta-regression p = 0.214). Table 4 gives the reasons for drop outs stated by the trial author.

**Table 4:** Drop out reasons in trials of Oral Anticoagulation

Source	Reasons Given for drop out in the intervention and control group
^38 ^White 1989, US	Difficulty performing measurements, no reason given and changed physician
^29 ^Horstkotte 1998, Germany	No reasons given
^34 ^Sawicki 1999, Germany	Died, refused to participate, stopped warfarin therapy
^23 ^Beyth 2000, US	Physical limitations such as severe arthritis or decreased vision. Patients also preferred alternative control method, stopped warfarin therapy or referred to a nursing home. A number of patients also decline to participate
^24 ^Cromheecke 2000, Holland	Progressive visual impairment
^32 ^Kortke 2001, Germany	Difficulties with the device, travel cost to high, illness/psychological, difficulties, lack of support form physician, illness or death in the family, lack of interest and preference for family physician
^35 ^Sidhu 2001, UK	Patients declined training due to distance from home and lack of confidence in technique. Difficulty obtaining samples, preference for general practitioner management and technical problems with the instrument
^25 ^Fitzmaurice 2000, UK	Did not attend training sessions, failed training assessment, loss of confidence in self – management and problems with manual dexterity.
^27 ^Gadisseur 2003, Holland	Patients could not find the time for training, excluded during training and not agreeing with the randomization process
^28 ^Gardiner 2004, UK	Poor compliance, serious illness, failure to attend training, visual problems, poor dexterity, difficulty obtaining sample, moved to another area and patient death
^31 ^Khan 2004, UK	Unable to self-monitor, discontinued warfarin before study completion
^36 ^Sunderji 2004, Canada	Stopped warfarin therapy, withdrawal of consent, difficulty with device, preference for physician management and adverse events
^33 ^Menendez-Jandula 2005, Spain	Declined before training mainly due to lack of self confidence, unable to cope with self-management and could not pass training course
^37 ^Voller 2005, Germany	No reasons given
^26 ^Fitzmaurice 2005, UK	Lost to follow up with no reason, withdrew consent, withdrawn at training stage, did not attend training, adverse event, discontinued warfarin, moved out of the area, and death
^30 ^Katz Unpublished, US	No drop outs reported

§ Coumatrack Monitor ‡ Coagucheck System ¤ Pro time Microcoagulation system ACC: Anticoagulation Clinic Care PCP: Primary Care physician managed SM: self adjusted treatment

**Figure 4:**
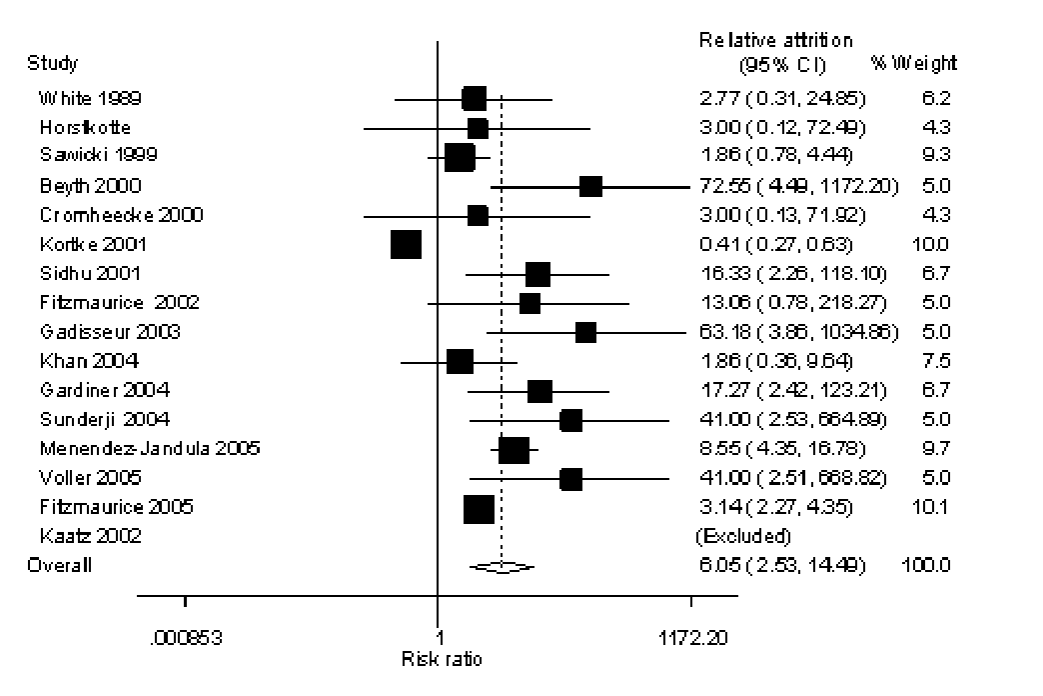
Oral anticoagulation attrition Forest plot.

Five trials report a RA higher than the upper limit of the 95% CI, equal to a higher than expected attrition in the intervention group, (figure 4); in comparison, only one (Kortke) [32] reported a RA below than the lower limit of the 95% CI. In this trial patients were assigned to the intervention directly after mechanical heart valve surgery. 90 patients were excluded from the analysis due to either post operative mortality or dropped out in the follow-up phase.

Of these five trials with higher RA one studied less than a 100 individuals: the Fitzmaurice trial [25] set in six general practices gave patients two training sessions and assessed competency for self-testing. Common reasons in this trial for exclusions pre-randomization were manual dexterity (13%), anxiety (12%), too elderly (12%), physically unwell (8%) and lack of cognitive ability (8%). During the study failure to attend training was the most frequent reason for withdrawal. Three trials [23,27,37] had similar drop outs in the intervention groups (range 19.2%–22.1%) and no drop outs in the control group. Beyth [23] recruited and trained hospitalized patients 65 years of age or older, 31 patients refused the intervention post randomization. Of 720 patients approached in the Gaddiseur trial [27] – set in anticoagulation clinics – 536 refused or were ineligible or unavailable. Of these 33% preferred existing system, 25% were too old, nervous or uncertain and 30% had no time or were not interested. Common reasons for drop outs were failing the training, problems with self dosing and differences greater than 20% between laboratory measures and the self testing device. The Voller trial [37] was discontinued due to poor recruitment, and the group comparisons were confined to an analysis of the INR measurements. Sunderji [36] recruited patients from a tertiary care institution or by referral as an outpatient. Clinical pharmacists and physicians selected patients on their assessment of competence, compliance and willingness to manage their own therapy. Despite this selection process 29% of the intervention group discontinued self testing compared to no drop outs in the control group: drop out reasons included difficulty with the monitor and a preference for physician management.

Three trials had very low drop out rates overall. [24,29,30] Katz unpublished trial [30] was set in anticoagulation clinics in a large integrated health system – mean age 63 years. Pre randomization patients had to prove they could schedule and attend anticoagulation clinics. A number found attending the anticoagulation clinics was geographically inaccessible. Patients phoned in results and were regularly contacted for telephone interviews. Horstkotte [29] recruited 150 consecutive patients directly following mechanical heart valve surgery. Cromheecke [24] study was a randomized crossover between self-adjusted treatment and anticoagulation clinic care.

Of the two largest trials [26,33] Menendez [33] had fewer drop outs (21.5% vs. 41.5%). Patients received three months of anticoagulation pre-randomization and did not have severe medical or physical illness. Of the 368 patients randomized to self management 58 declined before training, mostly because of a lack of confidence. Of those who received training ten could not pass and 11 dropped out post training. The Fitzmaurice trial [26] recruited unselected patients from general practice. Patients had to have a long term indication for anticoagulation and had at least six month of therapy. Of the 337 allocated to the intervention 95 did not receive it; mainly withdrawing at the training stage. A further 45 discontinued the intervention post training, the main reason being the patients' decision. The Fitzmaurice trial was the only paper to report the mean age of those who dropped out. The mean age of those invited to participate was 69 years (range 18–95) compared with a mean of 65 years for those recruited to the study. In the intervention group the mean age of those completing training was significantly lower than that of those initially randomized 61 yrs v 64 yrs (p = 0.012).

## Discussion

Pre-randomization drop-outs affect the external validity of a study while post-randomization drop-out affects internal validity, resulting in study bias. The presence of statistical heterogeneity in the analysis of relative attrition identifies interventions that are potentially less generalisable than others.

Therefore, analysis of attrition rates provides a wealth of information over and above that of the assessment of biasing the outcomes. For instance, RA was greater for self monitoring of oral anticoagulation than for diabetes. Potential reason for the increased RA for self-monitoring oral anticoagulation include: trials being larger and of greater duration; also many patients who self-monitored already had experience of an alternative management strategy and potentially preferred this system or to stay with it in the first place. In one trial where patients went straight on to self-monitor [32] and had no experience of usual management drop out rates were relatively low. In diabetes no alternative comparable testing strategy exists apart from comparison to urine testing, when one trial assessed patient preference, 71% preferred urine testing over SMBG. [15] In addition control group care was not always comparable to intervention care, for instance, control groups were often not provided training and therefore could not fail to attend a session that the intervention group could drop out of. Therefore additional elements of the intervention can act to increase relative attrition. Patients recruited to managed care programmes [12] or integrated health systems [30] may reduce drop outs. Individual training used in trials of anticoagulation [26] may not fair as well as group training; peer support may offer improved benefits over and above individual training. Possible reasons include the extra support for individuals within these systems of health care. It is worth noting for diabetes that training by general practitioners may result in excessive drop outs. [4] In addition the requirement to perform many dose adjustments; may increase anxiety and the possibility that self-testing in this area is difficult to perform.

Reported reasons for individuals not undertaking or completing self-monitoring were poor dexterity, anxiety, too elderly, concurrent illness and lack of cognitive ability. We consider that age should not be a restriction to self-monitoring, however in the one trial [26] that used an unselected population and reported the mean age of the population attrition, drop out rates were higher, the age of participants who successfully self-monitored was younger than those who initially entered the trial. Whether the major factor here was physician reluctance to continue with self-monitoring or the patient decision cannot be clarified by the current paper. However, where age affects conditions such as frailty, dexterity or visual impairment then as a co-factor it becomes a restriction to self-monitoring.

Of interest some trials excluded those unable to attend the training or set distance limits due to the control group treatment and trial monitoring required. [33] Thus these trials potentially excluded those most likely to benefit from self-monitoring.

Finally, despite attempts by clinical pharmacists and physicians attempts to assess patients' competence, compliance and willingness to manage their own therapy attrition remained high [36] therefore further research should focus on effective assessment and targeting of self-testing.

There are limitations to the data we have presented. These are mainly due to under reporting of the reasons for dropping out of the trial from both arms. Thus we could not test for the interaction effects. However to further this area of research we are planning an individual patient data meta-analysis and collecting further drop out data, in addition to clinical outcomes.

## Conclusion

In conclusion this paper demonstrates the use of relative attrition as a new tool in systematic review methodology which has the potential to identify patient, intervention and trial characteristics which may influence attrition in trials of self-monitoring of oral anticoagulation and diabetes. The method of relative attrition we present has the potential to be applied to other systematic reviews besides self-monitoring. It can be applied to both non-drug and drug interventions to elicit the reasons for attrition. The main limitation will be effective reporting of drop-outs in randomized trials.

## Competing interests

The author(s) declare that they have no competing interests.

## Authors' contributions

CH and RP conceived of the study. RP, CH, and PG had input to the statistical analyses. AW and EM contributed to the data extraction. DM had intellectual to the initial drafts of the manuscript. All authors contributed to the draft of the manuscript, approved the analyses and read and approved the final manuscript.

## Pre-publication history

The pre-publication history for this paper can be accessed here:


